# Physical activity and marital satisfaction among teachers: a mediation model of self-esteem, spirituality, and psychological distress

**DOI:** 10.3389/fspor.2026.1782014

**Published:** 2026-04-17

**Authors:** Haifa Snani, Hela Snani, Amayra Tannoubi, Hela Znazen, Abdullah Hamed Alliheibi, John Elvis Hagan, Fairouz Azaiez

**Affiliations:** 1 Higher Institute of Sport and Physical Education of Ksar-Said, Universite de La Manouba, Ksar Said, Tunisia; 2 Higher Institute of Sport and Physical Education of Gafsa, University of Gafsa, Gafsa, Tunisia; 3 Sports Performance Optimization Research Laboratory (LR09SEP01), National Center for Sports Medicine and Science (CNMSS), Tunis, Tunisia; 4 Department of Sport Health, College of Sport Sports Science and Physical Activity, Princess Nourah Bint Abdulrahman University, Riyadh, Saudi Arabia; 5 Department of Physical Education, College of Sport Sciences and Physical Activity, King Saud University, Riyadh, Saudi Arabia; 6 Department of Health, Physical Education and Recreation, University of Cape Coast, Cape Coast, Ghana; 7 Neurocognition and Action-Biomechanics-Research Group, Faculty of Psychology and Sports Science, Bielefeld University, Bielefeld, Germany; 8 Research Laboratory Education, Motricity, Sport Health, LR19JS01, High Institute of Sport and Physical Education of Sfax, University of Sfax, Sfax, Tunisia

**Keywords:** body esteem, marital satisfaction, physical activity, psychological distress, sexual self-esteem, spirituality, teachers, Tunisia

## Abstract

**Background/objectives:**

Marital satisfaction is influenced by various intrapersonal and environmental factors. The present study explores the associations between physical activity and marital satisfaction in a sample of Tunisian married teachers, examining the mediating roles of body esteem, sexual self-esteem, and psychological distress, with spirituality acting as a psychosocial resource.

**Methods:**

A cross-sectional study was conducted with 924 married teachers (52% women) from six Tunisian regions. Participants completed the Arabic versions of the Ironson-Woods Spirituality/Religiousness Index (IWSRI), Body Esteem Scale (BES), Sexual Self-Esteem Scale (SSES), Kessler Psychological Distress Scale (K10), and the Dyadic Adjustment Scale (DAS-4). The hypothesized pathways were examined using Structural Equation Modeling (SEM).

**Results:**

The Arabic IWSRI demonstrated strong psychometric properties (*α* = 0.961) with a confirmed four-factor structure. SEM revealed a significant positive association between physical activity and marital satisfaction (*β* = 0.34, *p* < 0.001) which was partially mediated by body esteem (*R*^2^ = 0.48), sexual self-esteem (*R*^2^ = 0.52), and reduced psychological distress (*R*^2^ = 0.24). Spirituality was positively correlated with both physical activity (*β* = 0.41, *p* < .001) and marital satisfaction (*β* = 0.29, *p* < .001). The overall model accounted for 63% of the variance in marital satisfaction.

**Conclusions:**

In this specific population of Tunisian teachers, physical activity is associated with higher marital satisfaction through both physiological and psychosocial dimensions. Spirituality appears to be a significant resource correlated with mental balance and resilience. These preliminary results support an integrative view of health—encompassing physical, psychological, and spiritual facets—for understanding factors linked to stable marital relationships. However, due to the cross-sectional design, causal directions cannot be inferred.

## 1 Introduction

The pursuit of meaning is the basic principle of human psychological functioning. From such a viewpoint, spirituality can be viewed as the most essential multidimensional construct that enables individuals to move through sociocultural or environmental factors; in other words, it embodies an internal regulatory force for promoting psychosocial adjustment and identity balance. Spirituality is a multifaceted construct representing the search for meaning (1, 2), transcendence (3), and a connection to the sacred (4). Its relevance in the humanities and social sciences has been firmly established (5, 6), revealing a robust relationship between spiritual beliefs, quality of life, and self-concept (7). Such beliefs often provide critical psychological sustenance during life's struggles (8), fostering hope and maintaining psychological equilibrium (9). Conversely, neglecting this inner sphere can hinder the achievement of a fulfilled life.

This internal equilibrium, however, does not exist in a vacuum; it is deeply intertwined with physical practices. Regular physical activity acts as a “protective factor” against internal distress, including anxiety and depressive symptoms (10, 11). Recent findings by Nie et al. (12) suggest that exercise promotes psychological homeostasis by relieving stress, enhancing emotional regulation, and refining the self-concept. At its core, physical activity buffers daily stressors, largely through the enhancement of self-esteem, a major determinant of global well-being (13).

Specifically, physical exercise contributes to a positive body image and reduces body dissatisfaction (14). Emerging evidence indicates that the mental benefits of exercise are often driven by subjective perception of one's appearance rather than the activity alone. A healthy connection with the body strengthens these benefits—a finding supported by Chen et al. (15), who demonstrated that higher self-valorization significantly increases daily well-being. This confidence extends to sexual self-esteem (16), which is crucial for a satisfying sexual life (17).

These intrapersonal resources significantly influence marital relationships. Marital satisfaction often relies on robust self-esteem and a fulfilling sexual life (18), which act as mutually reinforcing factors (19). Conversely, emotional unrest and psychological strain can undermine dyadic peace. Mental health issues or sexual dissatisfaction in one partner frequently correlate with diminished marital satisfaction (20, 21). This can trigger a vicious cycle where marital discord exacerbates emotional distress, further intensifying affective conflict (22, 23).

Within sport and exercise psychology, physical activity is increasingly conceptualized not only as physiological behavior but also as a psychosocial regulator influencing emotional functioning, self-perception, and interpersonal relationships (24, 25). Exercise participation has been linked to improved self-esteem, reduced psychological distress, and enhanced relationship satisfaction through mechanisms such as emotional regulation, body perception, and psychological resilience (25, 26). Understanding these pathways is particularly relevant for populations exposed to high occupational stress, such as teachers, for whom physical activity may function as a behavioral coping strategy that promotes both individual well-being and relational stability (24).

While research has explored the links between physical activity, mental well-being, and marital satisfaction, few studies have integrated the spiritual dimension into a comprehensive model. Spirituality is a structural cornerstone in many Arab societies, forming the crux of daily life (27). Accordingly, the present study first validates the Arabic version of the Ironson-Woods Spirituality/Religiousness Index (IWSRI) (28) among a sample of teachers in Tunisia. Using Structural Equation Modeling (SEM), this research aims to examine whether psychosocial assets—specifically physical and sexual self-esteem and the reduction of distress—mediate the association between physical activity and marital satisfaction. Finally, we examine how spirituality, acting as a global psychosocial resource, directly and indirectly contributes to physical activity engagement and marital satisfaction within an integrative mediation framework. By analyzing these complex pathways, the study provides a holistic understanding of the determinants of marital welfare and human flourishing.

## 2 Materials and methods

### 2.1 Participants

In the current study, we recruited 924 teachers from various academic disciplines to validate the Arabic version of the Ironson-Woods Spirituality/Religiousness Index (IWSRI). The sample totaled 440 men and 484 women from six zones of Tunisia: North-East, North-West, Center-East, Center-West, South-East, and South-West. To ensure the reliability of the translated version, a group of 50 participants were sampled using a test-retest procedure. Primary, secondary, and higher education teachers employed in such a facility had at least one year of experience in professional practice and were the target population. Participants were recruited via convenience sampling from various educational and university establishments. Inclusion criteria were: (i) 25–60 years old, (ii) married, and (iii) volunteer informed consent. Exclusion criteria comprised: (iv) unstable marital status (e.g., separation, divorce or bereavement), (v) a serious medical condition or diagnosis of severe psychiatric disorder and (vi) current pregnancy. Questionnaires with more than 5% of missing data and inconsistent responses were also excluded from the final analysis.

### 2.2 Procedures and ethical statement

Cross-cultural adaptation was performed in a rigorous protocol where a series of translations of the index was implemented back and forth from the source language and into the target language. It helps to catch errors and to clarify unclear items. Two different teams carried out the translation. The initial team were translators who experienced the questionnaire, both in its clinical uses. The second team would consist of people who had no prior knowledge of the humanities or of the study's goals, ensuring translation that was in the native language of the population in question, reducing academic bias. This approach is consistent with existing international standards for transcultural validation of psychological instruments (46, 47). Ethical approval for this study was granted by the Ethics Committee of the Sousse University Hospital on March 21, 2022 (Ref: CEFMS 113/2022). Following this approval, written informed consent was obtained from all participants prior to their involvement. To ensure clarity and support, the questionnaires were administered in small groups. This process was facilitated by two trained research assistants—both post-graduate professionals—who were present to address any questions and ensure the participants felt comfortable throughout the data collection process.

### 2.3 Instrument

The Ironson-Woods Spirituality/Religiousness Index [IWSRI (28)]. The brief version of IWSRI (short form) has 25 items including two subscales; spirituality and religiousness—both are measured in the IWSRI subscale format. The latter was adapted from the original 89 item scale that was too cumbersome for practical applications. The concept of spirituality was identified using the IWSRI, which compares religiosity in health assessment and spirituality as concepts both together and separately, also public health influences to be researched. It is a cognitive-behavioral assessment consisting of four dimensions: (i) Sense of Peace (Items 1–9): peacefulness, spiritual comfort, meaning and connection; (ii) Faith in God (Items 10–15); (iii) Role of God in Recovery (Items 16–20); and (iv) Compassion toward Others (Items 21–25): respect, tolerating, connecting with others. Answers are scored on a 5 point scale; higher scores indicate higher levels of spirituality or religiousness.

Physical activity self-assessment (29): The Ricci & Gagnon physical activity tool measures a person's global level of activity. It is commonly used in health and sports-sciences studies to classify individuals based on their weekly activity. The 9-item questionnaire assessed sedentary behavior, leisure/sports activities (4 items), and daily tasks (4 items). Scores range from 9 to 45, where a total score <18 signifies inactivity, 18–35 indicates an active lifestyle, and >35 denotes a very active individual. In the current study, the total continuous score was used as a manifest variable (observed variable) within the Structural Equation Modeling (SEM). The internal consistency for the Ricci & Gagnon scale in this sample was satisfactory, with a Cronbach's alpha of 0.78.

Physical Well-Being: Body Esteem Scale (BES; 48) The BES is a well-established research instrument measuring how well an individual values their body. It assesses satisfaction with multiple dimensions of body image, including aesthetic, fitness, and general appearance, giving it a 360-degree perspective. Items are rated on a 5-point scale, rating satisfaction in dimensions like attractiveness and weight concern. Reliability of the scale is very high; in many cases Cronbach's alpha was also higher than 0.85.

Dyadic Adjustment Scale [DAS-4 (30)]. The DAS-4 is an adapted version of the Dyadic Adjustment Scale to determine relationship quality. There are 4 items focusing on dyadic agreement, satisfaction, cohesion and emotional expression. For all its brevity, however, it has high sensitivity in finding signs of relational distress. Each item is rated on a 5- to 6-point Likert scale. A total score below 13 indicates distress within the partnership. Its internal consistency score is excellent (*α* = 0.89 for women, *α* = 0.78 for men).

Sexual Self-Esteem Scale [SSES (31)]. Sexual self-esteem is described as an individual's appraisal of his/her own worth, competence and satisfaction regarding their sexuality. It is a central element of relational self-esteem. According to the 5-point Likert scale (from “strongly disagree” to “strongly agree”), the SSES assesses: (i) sexual confidence, (ii) personal feelings of competence and attractiveness, (iii) positive experience of sexual relationships, and (iv) ease and satisfaction during intimate relationships.

Psychological distress Kessler was measured with the Kessler Psychological Distress Scale [K10 (20)]. Psychological distress is defined as a mental state, that is, a state of depression or anxiety. The French-language version used in the Canadian Community Health Survey was employed in this study. Respondents were asked how often they had been unhappy or depressed over the past 30 days. Responses are recorded on a 5-point Likert scale from 0 to 4: none of the time, a little of the time, some of the time, most of the time, and all the time. Scores can range from 0 to 40, with higher scores meaning more psychological distress. Although measured with an ordinal type of scale, these scores are taken as ratio data for statistical analysis. The K10 has been validated in the past in groups of French-speaking people.

### 2.4 Data analysis

To ensure robust construct validation of the Arabic version of the Ironson–Woods Spirituality/Religiousness Index (IWSRI), a split-sample validation strategy was employed (32, 33). The total sample (*N* = 924) was randomly divided into two independent subsamples. The first subsample (*n* = 462) was used to conduct the Exploratory Factor Analysis (EFA) to identify the underlying factorial structure of the instrument. The second subsample (*n* = 462) was subsequently used to perform the Confirmatory Factor Analysis (CFA) to test the stability and adequacy of the factor structure identified in the exploratory phase. This two-step approach follows recommended psychometric validation procedures and reduces the risk of model overfitting when exploratory and confirmatory analyses are conducted on the same dataset (34, 35).

We conducted all statistical analyses with commercial software “Statistical Package for the Social Sciences” (IBM SPSS software for Windows, version 26.0, IBM Corp., Armonk, NY), whereas we conducted the CFA with commercial software “Analysis of a moment structures” (IBM Corp., Armonk, NY). (Amos software for Windows, version 23.0, IBM, SPSS, Chicago, United States).

Unweighted least squares (ULS) extraction with oblique Oblimin rotation was applied, allowing correlations among latent dimensions of spirituality, and Kaiser normalization was used to estimate the factorial loads of the items in this study, and items with factorial loads less than 0.4 were removed. The reliability of the instrument was examined by the Cronbach coefficient *α*. A Cronbach's *α* above the threshold of 0.70 was considered acceptable, above 0.80 as good, and between 0.90 and 0.95 as excellent). The questionnaire structure for the entire population was carried out by confirmatory factor analysis (CFA). To evaluate the model, many CFA indices were used: (a) the 2; (b) the 2/DF; (c) the Comparative Fit Index (CFI); (d) the Tucker–Lewis Index (TLI); (e) the Standardized Root Mean Square Residual; and (f) the Root Mean Square of error Approximation (RMSEA).

Hu and Bentler (36) recommended that the CFI and TLI be more than 0.95, and that the RMSEA be less than 0.08 for fair revisions. Because only self-reported measures were used, the possible effect of common method variance was investigated. Because all constructs were measured using self-report questionnaires administered at a single time point, the potential influence of common method bias (CMB) was examined. First, Harman's single-factor test was conducted using exploratory factor analysis. The results indicated that the first unrotated factor accounted for less than 40% of the total variance, suggesting that common method bias was unlikely to represent a serious threat. In addition, procedural remedies were implemented during data collection, including assuring participant anonymity, emphasizing the absence of right or wrong answers, and varying scale formats across questionnaires. These precautions reduce evaluation apprehension and minimize method bias in survey research (37).

To formally test mediation effects, indirect effects were estimated using a bias-corrected bootstrap procedure with 5,000 resamples, as recommended for mediation analysis in structural equation modeling. Bootstrapping provides a non-parametric estimation of the sampling distribution of indirect effects and generates 95% confidence intervals (CI). Indirect effects were considered statistically significant when the confidence interval did not include zero (38, 49)

## 3 Results

### 3.1 Sociodemographic characteristics of respondents

Survey respondents in a Sociodemographic Context. The final study sample population was 924 participants (Table 1); 52.38% were women and 47.62% were men (sex ratio: 0.91) and they also come from the diverse regions of Tunisia. The total average age of the participants was 42.37 ± 7.207 years.

**Table 1:** Characteristics of the study population.

Variable	Category	Male		Female		Total	
		Frequency (*N*)	%	Frequency (*N*)	%	Frequency (*N*)	%
Sex		440	47.62	484	52.38	924	100
Age		440 $\bar{x}=43.90$ (***σ*** ± 6.714)		484 $\bar{x}=40.99$ (***σ*** ± 7.365)		924 $\bar{x}=42.37$ (***σ*** ± 7.207)	
Physical Activity Level	Inactive	148	16.02%	128	13.85%	275	29.87%
	Active	292	31.60%	356	38.53%	648	70.13%
Marital Duration	Less than 5 years	64	6.9%	60	6.5%	124	13.42%
	5 to 10 year	146	15.8%	126	13.6%	272	29.44%
	11 to 15 years	89	9.6%	149	16.1%	238	25.76%
	16 to 25 years	106	11.5%	112	12.1%	218	23.59%
	More than 25 years	35	3.8%	37	4.0%	72	7.79%
Body Mass Index	Underweight (<18)	7	0.8%	6	0.6%	13	1.41%
	Normal weight (18–25)	165	17.9%	230	24.9%	395	42.75%
	Overweight (26–30)	217	23.5%	150	16.2%	367	39.72%
	Moderate Obesity (31–40)	49	10.3%	95	10.3%	144	15.55%
	Severe Obesity (>40)	2	0.2%	3	0.3%	5	0.54%
Dependent Children	No children	52	5.62%	42	4.55%	94	10.17%
	1 to 2 children	248	26.84%	294	31.82%	542	58.66%
	3 or more children	140	15.15%	148	16.02%	288	31.17%
Place of Residence	North-East	175	19%	194	21.0%	369	39.72%
	North-West	50	5.4%	24	2.6%	74	8.01
	Center-East	173	18.8%	222	24.1%	395	42.75%
	Center-West	23	2.5%	17	1.8%	40	4.33%
	South-East	10	1.1%	16	1.7%	26	2.81%
	South-West	8	0.9%	10	1.1%	18	1.95%
Academic Disciplines)	Sciences	53	5.74%	98	10.61%	296	16.3%
	Literature	137	14.83%	189	20.46%	326	35.3%
	Technical Sciences	25	2.71%	17	1.84%	32	3.2%
	Computer Science	30	3.24%	13	1.41%	53	5.7%
	Physical Education	172	18.61%	124	13.42%	296	32.0%
	Economics and Management	23	2.48%	43	4.65%	66	7.1%

Regarding their family and marital history, the duration of marriage ranged from 1 to 45 years, with a mean of 13,27 ± 8,01 years. In household composition, 10.7 percent of the participants did not have dependent children, 58.66 percent were caring for one or two children and 31.17% had three or more children. The average Body Mass Index (BMI) for the participants was 26,19 kg/m^2^ (±3,94) indicating that the study population group is slightly overweight. In general, 42.75% were within the normal weight range, 39.72% were overweight, and 16.09% obese. Aside from their physical well-being, the research also emphasizes the considerable psychological and physiological pressures placed on these educators. In fact, at the time of the study, nearly one in three teachers (30.94%) attended a therapist to solve stress-related issues and anxiety. Moreover, 18.35% were consulting a physician for persistent headaches, and 16.55% were treated for infections of different kinds, highlighting their personal and business surroundings on their overall health status.

### 3.2 Sexual activity frequency

**Figure 1:**
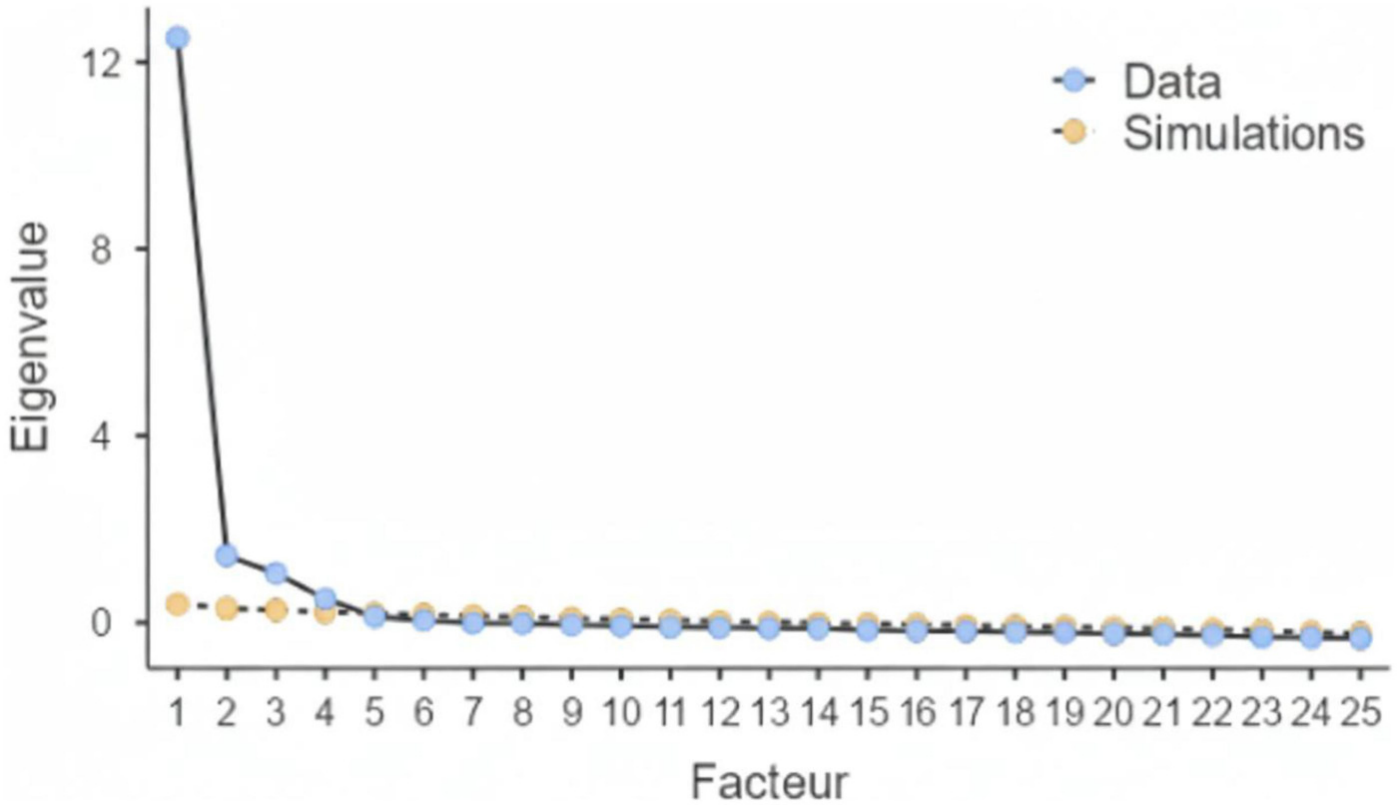
Scree plot confirming the ironson-woods spirituality/religiousness Index.

Most participants (51.2%) engaged in two sexual episodes per week, while 22.6 percent had one sexual encounter per week, and 26.2 percent had three or more sexual encounters according to Figure 1. Men reported significantly more sexual intercourse than women (*p* = 0.003). 32.7% of men indicated they have had at least three sexual encounters each week compared with 20.3% of women.

### 3.3 Exploratory factor analysis (EFA)

The psychometric quality of the construct was determined by orthogonal exploratory factor analysis using Varimax rotation in the 25-item questionnaire (28). To increase the readability of the factor-loading table, only the respective item loadings ≥0.30 were presented, as recommended by Archer and Haigh (39) and Tabachnick and Fidell (40). It was found that the Arabic version of the Ironson-Woods Spirituality/Religiousness Index [(IWSRI (28)] also provided great internal consistency (Cronbach's *α* = 0.961) and temporal stability with a test–retest reliability coefficient of *r* = 0.921. An exploratory factor analysis (EFA) was conducted on the first randomly selected subsample (*n* = 462) to examine the factorial structure of the Arabic version of the IWSRI.

All 25 items were included to assess the underlying dimensions of spirituality and religiousness. Sampling was tested for adequacy using the Kaiser–Meyer–Olkin (KMO) measure, obtaining a value of 0.960, above an acceptable benchmark (0.50) (33). Bartlett's test of sphericity was statistically significant (*χ*^2^ = 17,781.353, df = 300, *p* < 0.001), suggesting that the correlation matrix was suitable for factor analysis (41). The scree plot (Figure 1) with eigenvalues > 1 was explored to decide how many factors to keep. Furthermore, a parallel analysis was confirmed by a sample of 924 randomly simulated datasets to find a four-factor solution. The factors detected were Sense of Peace (SP), Faith in God (FG), Religious Behavior (RB), and Compassion toward Others (CO). In this way, all four factors accounted for 52.056%, 7.679%, 6.286%, and 4.060%, respectively, confirming the multidimensional framework of the Ironson-Woods Spirituality/Religiousness Index. Reliability Analysis. The reliability of the IWSRI dimensions was assessed by Cronbach's alpha and *ω* coefficients. The reliability of all four factors was found to be good to excellent (*α*/*ω* = 0.930, 0.910, 0.897, 0.918, respectively). Cronbach's alpha values > 0.80 indicate high inter–item correlations, with internal consistency being consistent across multiple measures and reliability of each dimension of the scale being robust.

**Figure 2:**
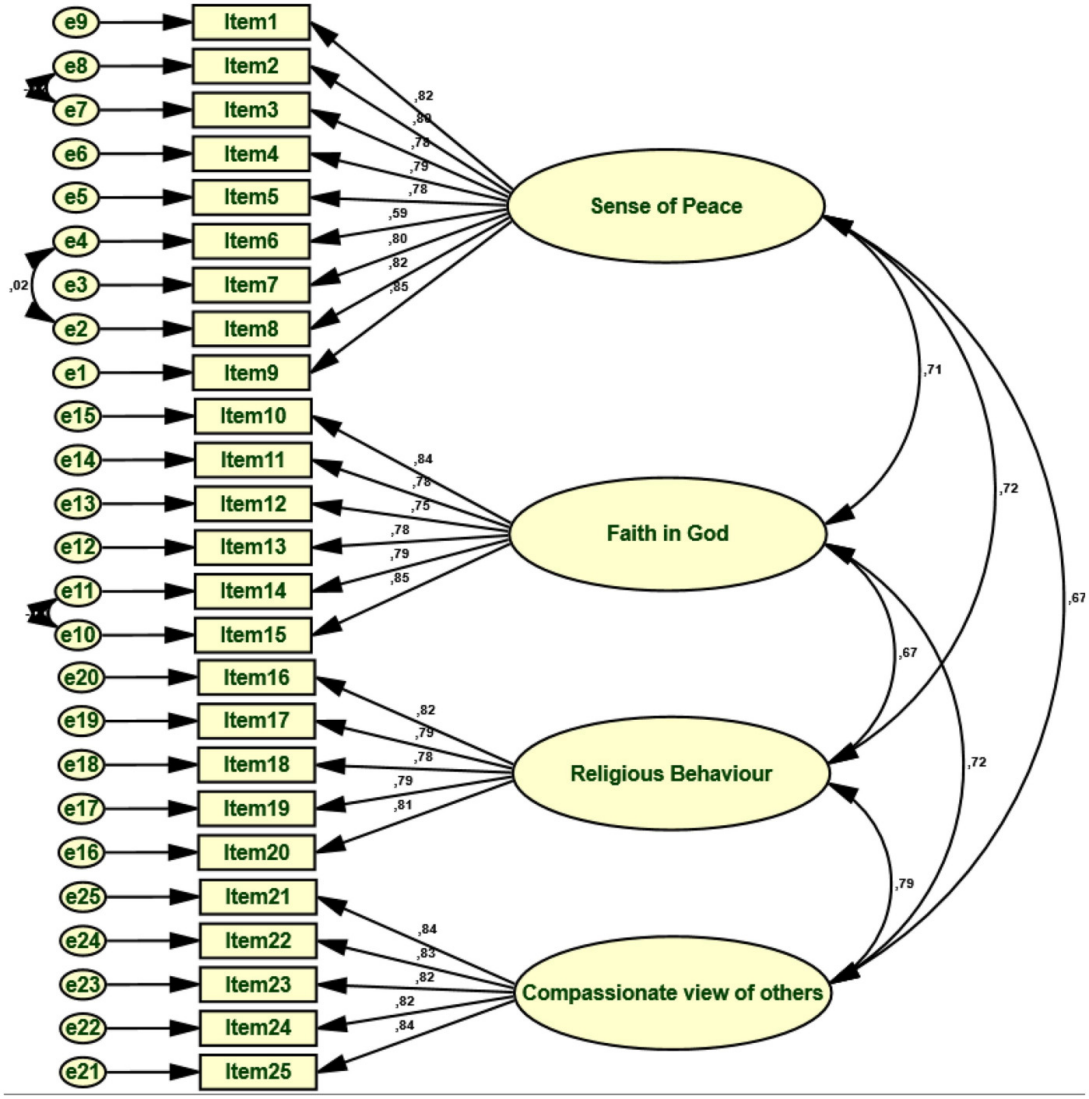
Standardized regression loadings of single factor CFA after covariance adding physical activity, body esteem scale, psychological distress, and marital satisfaction among teachers.

### 3.4 Factorial structure and variance

As a result of the tests a model is produced that includes four main components, which together cover 70.081% of the variance. Factor 1 (Eigenvalue = 13.04): The items associated with Sense of Peace (Items 1–9). Factor 2 (Eigenvalue = 1.920): Items associated with Faith in God (Items 10–15). Factor 3 (Eigenvalue = 1.571): Items are Religious Behavior related (Items 16–20). Factor 4 (Eigenvalue = 1.015): Items are Compassion toward Others (Items 21–25). The eigenvalue analysis is presented in Table 2 when we break down the factor loadings of the IWSRI items into four components. With their respective factors, they all have very good associations showing that there is a four-factor structure for the Arabic-IWSRI model. Uniqueness and Item Representation Analysis of uniqueness indices (Supplementary Appendix A) reveals that most items reflect less than 0.40, meaning that the selected factor solution has good representation. Only one item showed significant uniqueness (u^2^ = 0,638), indicating its variance may be largely unexplained by the factors extracted. Theoretical significance of this item kept on this item for now is treated separately in the discussion. In summary, these findings reinforce the validity and reproducibility of the factorial structure of the questionnaire.

**Table 2:** Inter-factor correlations of the ironson-woods spirituality/religiousness Index (IWSRI).

Correlations	Sense of Peace	Faith in God	R. Behaviour	Compassionate
Sense of Peace	1			
Faith in God	.672^a^	1		
Religiuos Behaviour	.661^a^	.613^a^	1	
Compassionate view of others	.628^a^	.671^a^	.721^a^	1

^a^ The correlation is significant at level 0.01 (bilateral).

### 3.5 Convergent and discriminant validity

Correlations among dimensions ranged moderately to high, from 0.67 to 0.79 (Table 2). These coefficients imply that the constructs are related but not redundant, which supports the discriminant validity of the model (50). As a result, the Spiritual Experience appears as an integrated multifactorial structure, comprising emotions, beliefs, religious beliefs, and social relations. Overall findings support the construct validity of the questionnaire. The theoretical factorial framework is established, the internal consistency is adequate, and the inter-component relationships are conceptually coherent. As a result, considering theoretical and practical considerations, the instrument will be considered as psychometrically valid for investigating spirituality in the chosen context.

### 3.6 Composite reliability and average variance extracted

The Composite Reliability (CR) values are generally satisfactory, with most dimensions displaying coefficients above the recommended threshold of 0.70, indicating robust internal consistency. While several Average Variance Extracted (AVE) values are below 0.50, they remain acceptable given that the composite reliability is adequate. According to the recommendations of Fornell and Larcker (42), these results support an overall satisfactory convergent validity for the measurement model. Methodological guidelines suggest that when AVE is below 0.50 but CR exceeds 0.70, convergent validity can be considered adequate, particularly in the social and human sciences where constructs are complex and multidimensional (42, 51)

### 3.7 Confirmatory factor analysis (CFA)

The confirmatory factor analysis (CFA) was conducted on the second independent subsample (*n* = 462) to test the factorial structure identified in the exploratory analysis. This cross-validation procedure enhances the robustness and generalizability of the measurement model.

The model derived from the Confirmatory Factor Analysis (CFA) demonstrates a very good fit with the empirical data (Table 3). The *χ*^2^/df ratio (1.203), which is below the recommended threshold of 3, indicates a satisfactory model fit, despite the known sensitivity of the *χ*^2^ test to large sample sizes (43). Absolute fit indices show satisfactory to excellent values (GFI = .904; AGFI = .883; RMR = .038), indicating low average residuals and a good fit for the observed covariance matrix (33). Furthermore, incremental fit indices (NFI = .928; CFI = .987; IFI = .987; TLI = .985) significantly exceed the recommended thresholds of.90 and even.95, confirming the superiority of the tested model relative to the null model (36, 44). Finally, the low RMSEA value (0.030) reflects high-quality model-population approximation, suggesting a stable and parsimonious factor structure (45). The RMSEA point estimate is supported by a 90% confidence interval ranging from.015 to.041, with a PCLOSE of.999, indicating a high probability that the model has minimal approximation error. Taken together, these results demonstrate that the evaluated model is statistically robust, theoretically coherent, and well-fitted with the empirical data.

**Table 3:** Confirmatory factor analysis (CFA) Fit indices for the ironson-woods spirituality/religiousness Index (IWSRI).

Indices	*χ* ^2^	df	χ^2^/df	GFI	NFI	AGFI	RMR	CFI	IFI	TLI	RMSEA
Model	320.038	266	1.203	.904	.928	.883	.038	.987	.987	.985	.030

*N* *=* 924*,* χ^2^, Chi-Square; df, Degree of Freedom; GFI, Goodness of Fit Index; NFI, Normed-Fit Index; AGFI, Adjusted Goodness-of-Fit Index; SRMR, Standardized Root Mean Square Residual; CFI, Comparative Fit Index; PNFI,: Parsimony Normal Fit Index; RMSEA, Root Mean Square Eroor of Aproximation.

We found that the 25-item model represents a better fit for our theoretical model for all indices within our population. The evidence of the four-dimensional factorial structure of the teacher population is confirmed by the validation of the Arabic Ironson-Woods Spirituality/Religiousness Index (Figure 2). In fact, the overall findings present strong evidence of construct validity of the questionnaire. The theoretical factorial structure is confirmed, internal consistency is acceptable, and the relationships between items are conceptually coherent. Hence, psychometrically this instrument is reliable for assessing spirituality in the selected context (Figure 2).

**Figure 3:**
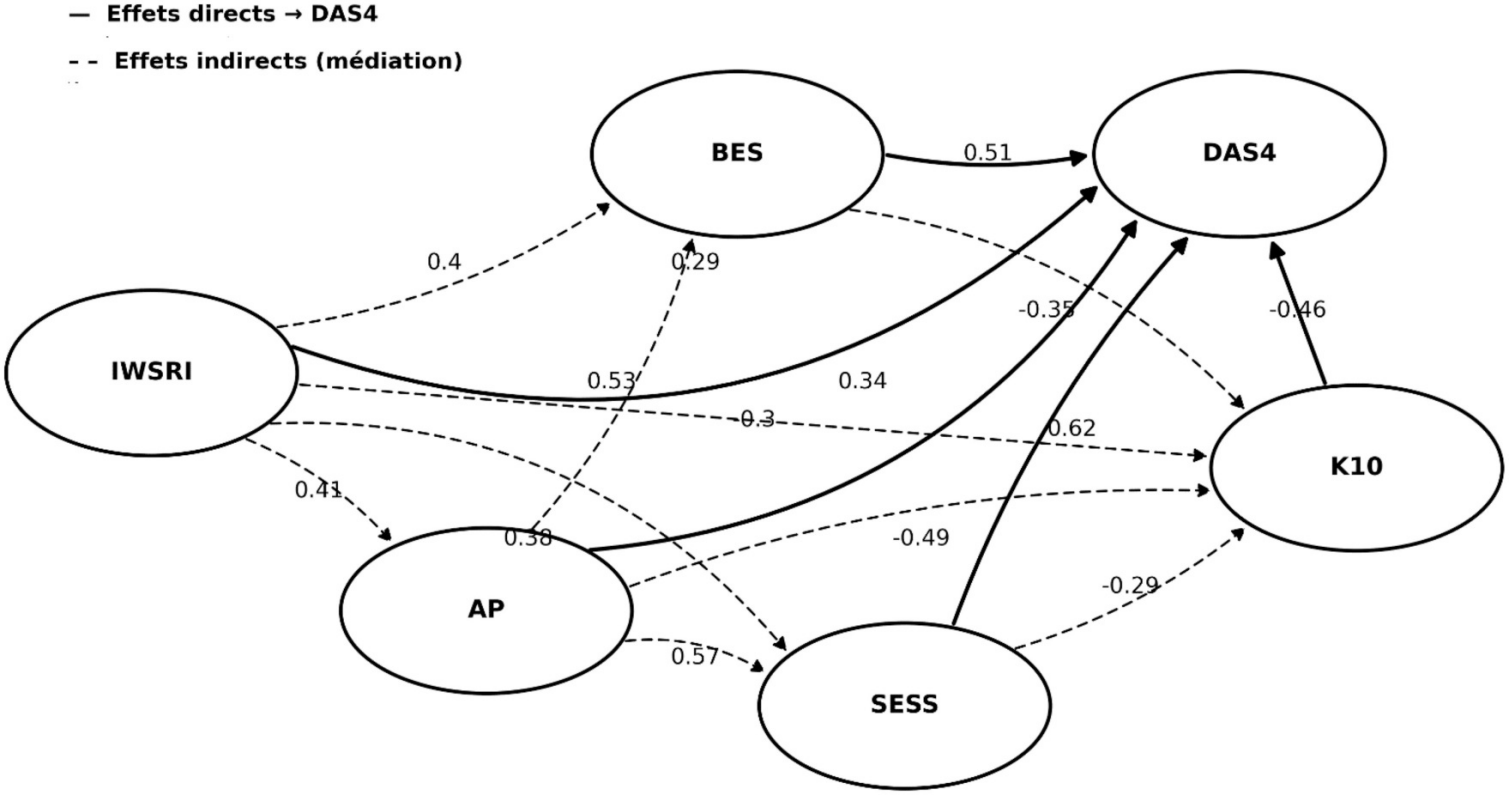
SEM model - visual hierarchy of effects. IWSRI: Spirituality; AP, Physical Activity; BES, Body Self-Esteem; SESS, Sexual Self-Esteem; K10, Psychological Stress; DAS4, Marital Satisfaction.

Figure 3 presents the tested structured mediation model, illustrating both direct and indirect pathways linking physical activity, spirituality, psychosocial resources, and martial satisfaction. Physical activity showed a direct positive association with marital satisfaction (*β = 0.34, p < .001*), which is consistent with its potential role among teachers. Spirituality is positively associated with physical activity (*β = 0.41, p < .001*), and body and sexual self-perception, hinting at a structuring link with behaviors and psychosocial resources. Physical exercise has an indirect association with marital satisfaction, through associations with body (*β* *=* *0.53, p* *<* *.001*), and sexual self-esteem (*β* *=* *0.57, p* *<* *.001*). Physical activity and self-esteem are associated with lower psychological distress, the latter of which has significant negative association with satisfaction (*β* *=* *−0.46, p* *<* *.001*). Mediation results suggest that the relationship between physical exercise and satisfaction in the marital relationship is partially mediated through self-esteem and psychological distress. The persistent direct association of spirituality with marital satisfaction indicates an integrative mediation combining both direct and indirect pathways. The model thus offers overall strong theoretical and statistical coherence, suggesting that psychosocial resources act as mediating variables in the nexus between physical activity and marital satisfaction.

Using structural equation modeling, our model demonstrates high explanatory power (Table 4). Spirituality shows a strong positive association with physical activity (*β* = 0.41, *p* < .001), accounting for 17% of its variance (*R*^2^ = 0.17). Spirituality (*β* = 0.40, *p* < .001) and physical exercise (*β* = 0.53, *p* < .001) are significantly linked to body self-esteem, with 48% of the variance explained (*R*^2^ = 0.48). Similarly, spirituality (*β* = 0.38, *p* < .001) and physical exercise (*β* = 0.57, *p* < .001) are significant correlates of sexual self-esteem (*R*^2^ = 0.52). Psychological distress is negatively associated with spirituality (*β* = −0.30, *p* < .001), physical activity (*β* = −0.49, *p* < .001), body self-esteem (*β* = −0.35, *p* < .001), and sexual self-esteem (*β* = −0.29, *p* < .001), with a moderate explained variance (*R*^2^ = 0.24). Lastly, marital satisfaction is significantly related to spirituality (*β* = 0.29, *p* < .001), physical activity (*β* = 0.34, *p* < .001), body self-esteem (*β* = 0.51, *p* < .001), sexual self-esteem (*β* = 0.62, *p* < .001), and psychological distress (*β* = −0.46, *p* < .001), with a high total explained variance (*R*^2^ = 0.63). Taken together, this evidence reveals direct and indirect pathways and supports a partial mediation model, where psychosocial resources mediate the associations between physical activity and teachers' marital satisfaction.

**Table 4:** Standardized direct effects (*β*) and explained variance (R^2^) of the SEM model.

Dependent variable (Endogenous)	Predictor	*β* (Standardized)	R^2^
Physical Activity (PA)	Spirituality (IWSRI)	0.41***	0.17
Body Self-Esteem (BES)	Spirituality (IWSRI)	0.40***	0.48
	Physical Activity (PA)	0.53***	
Sexual Self-Esteem (SESS)	Spirituality (IWSRI)	0.38***	0.52
	Physical Activity (PA)	0.57***	
Psychological Distress (K10)	Spirituality (IWSRI)	−0.30***	0.24
	Physical Activity (PA)	−0.49***	
	Body Self-Esteem (BES)	−0.35***	
	Sexual Self-Esteem (SESS)	−0.29***	
Marital Satisfaction (DAS4)	Spirituality (IWSRI)	0.29***	0.63
	Physical Activity (PA)	0.34***	
	Body Self-Esteem (BES)	0.51***	
	Sexual Self-Esteem (SESS)	0.62***	
	Psychological Distress (K10)	−0.46***	

*** *p* < .001. *β:* Standardized path coefficient; *R2*: Coefficient of determination.

As shown in Table 5, bootstrapping analyses confirm the statistical significance of the indirect pathways. The indirect effect of physical activity on marital satisfaction through body self-esteem was significant [*β* = 0.27, 95% CI (0.19, 0.35)]. Similarly, sexual self-esteem significantly mediated the relationship between physical activity and marital satisfaction [*β* = 0.35, 95% CI (0.26, 0.44)]. Psychological distress also served as a significant mediator [*β* = 0.23, 95% CI (0.16, 0.31)]. Because the direct path between physical activity and marital satisfaction remained significant (*β* = 0.34, *p* < .001), the results support a partial mediation model.

**Table 5:** Bootstrapped indirect effects of physical activity on marital satisfaction.

Indirect Path	Standardized Effect	95% CI Lower	95% CI Upper	Result
PA → Body Self-Esteem → Marital Satisfaction	0.27	0.19	0.35	Significant
PA → Sexual Self-Esteem → Marital Satisfaction	0.35	0.26	0.44	Significant
PA → Psychological Distress → Marital Satisfaction	0.23	0.16	0.31	Significant
Total Indirect Effect	0.85	0.71	0.99	Significant

## 4 Discussion

Overall, the results reveal rather satisfactory composite reliability with most constructs having acceptable CR values, indicating a good internal consistency (33). While several AVE values are lower than 0.50, when CR is greater than 0.60, convergent validity is satisfied, which according to Fornell and Larcker (42) would be acceptable. Such a profile is typical of the complexity of multidimensional psychosocial models (46). Accordingly, the measured indices further contribute to the robustness of the measurement model, which justifies the use for structural analyses. Importantly, the construct validity of the spirituality scale was strengthened through a split-sample validation procedure, where exploratory and confirmatory factor analyses were conducted on two independent subsamples. This methodological strategy reduces the likelihood of capitalization on chance and provides stronger evidence for the stability of the measurement model.

Overall, the indices validate the model's psychometric robustness and allow reading of the structural relations. Regarding the psychometric properties of the instruments used in this study, the Exploratory Factor Analysis (EFA) revealed that one item exhibited a high uniqueness (*u*^2^ *=* *0.638*). Although this item was retained to maintain the theoretical integrity of the scale, its high specific variance suggests that it may capture dimensions not fully accounted for by the primary factor within this specific population of Tunisian teachers. This result could be attributed to cultural nuances or potential linguistic subtleties in the translated version of the questionnaire, which might have influenced how participants perceived this specific task. Nevertheless, the overall structural model remained robust, and the inclusion of this item did not significantly alter the observed relationships between physical activity and psychological well-being.

Our SEM model indicates that physical activity is significantly associated with psychological well-being, aligning with emerging evidence that underscores the complex mechanisms through which exercise supports emotional health. Specifically, our findings suggest that physical activity may reduce psychological distress through direct pathways as well as through key psychological mediators, such as emotion regulation and self-perception (12). This is consistent with recent literature indicating that self-esteem and body image act as vital mediators between physical activity and quality of life, suggesting that fostering positive self-perceptions is a crucial mechanism in the link between exercise and mental health (15). Furthermore, a recent systematic review by White et al. (11) provided strong evidence for the mediating role of self-esteem between physical activity and mental health, further supporting our findings that psychosocial resources serve as significant intermediaries. These results are also corroborated by psychological wellness models showing that self-esteem and personal identity function as partial mediators between physical activity and subjective well-being (52). Nevertheless, it is essential to note that due to the cross-sectional design of this study, these relationships should be interpreted as bidirectional associations rather than definitive causal pathways. From a sport and exercise psychology perspective, the present findings contribute to understanding how behavioral engagement in physical activity may influence broader psychosocial outcomes beyond individual mental health (24, 25). The results suggest that exercise may enhance relational functioning through mechanisms involving self-perception and emotional regulation, which are core constructs within sport psychology frameworks (26). These findings therefore extend previous research by demonstrating that the psychological benefits of physical activity may also operate at the interpersonal level, influencing marital satisfaction through psychosocial mediators (25).

These findings support the theoretical robustness of our integrated framework where spirituality, physical activity, and internal psychology resources are combined to determine marital satisfaction. Moreover, there is evidence in the literature to demonstrate associations between higher levels of physical activity and lower levels of symptoms of stress and anxiety (11) and confirm the negative impact psychological distress can have on well-being and social relationships. Finally, a few recent studies show that exercise can also alleviate distress through physiological and psychosocial aspects, that supports our findings that emphasize the relevant roles of psychosocial mediators in understanding the role of physical activity on marital satisfaction. Taken together, this recent accumulation of evidence demonstrates that exercising contributes positively via cognitive and emotional mechanisms, not limited to the physical, thereby providing additional support for the conceptual and empirical validity of our mediating model.

### 4.1 Limitations and viewpoints

Several limitations should be acknowledged. First, we are focusing on our analysis only cross-sectional in nature based. Data were collected at one point in time, which precludes establishing definitively causality. Confirming the directionality of effects over time would require longitudinal studies tracking the participants for multiple years. Second, our sample was convenient—it included readily and willingly identified participants. This decreases the generalization of the results to the general population. In a more representative sample, however, conclusions would be different. Third, we relied on self-administered questionnaires in which participants provided feedback on sensitive topics, like sexual satisfaction or spirituality, upon self-administration. This may inject bias, because people react socially to positive information rather than more brutally to it. To highlight the significance of a holistic approach to health, this work emphasizes an integrated perspective. It implies that one of the best strategies is linked to an active lifestyle while the other involves the psychological and spiritual level. This will lead to personal flourishing and could result in lifelong stability of couple relationships. Future longitudinal and dyadic studies that include partner reports and objective measures of physical activity would enhance causal inference and mitigate mono-method bias. In addition, although statistical checks suggested that common method variance was not dominant, the exclusive reliance on self-reported measures may still introduce shared method variance. Future studies should incorporate multi-method designs, such as partner reports or objective physical activity measurements (e.g., accelerometers), to further reduce potential bias.

## 5 Conclusions

The role played by physical activity is one of the central dimensions in the marital satisfaction of the studied Tunisian teachers, as supported by our structural equation model. The study shows that exercise is associated with psychological and spiritual resources, aside from physiological benefits, which are linked to the quality of the marital relationship. The research highlights the pathways through which routine physical activities relate to improved self-image and sexual self-esteem, as well as the regulation of psychological distress. Within this specific population, exercise is correlated with lower levels of anxiety and stress created by work and life demands, potentially contributing to a calmer relational environment. Moreover, spirituality as a coping strategy appears to be a framework associated with emotional well-being through both physical and psychological balance, thereby supporting the resilience of the couples studied.

## Data Availability

The raw data supporting the conclusions of this article will be made available by the authors, without undue reservation.

## Supplementary material

Supplementary Appendix A Uniqueness and item representation analysis of the 25 items of the Arabic version of the Ironson-Woods Spirituality/Religiousness Index (IWSRI).
