# Supplementary material for: Physical activity and marital satisfaction among teachers: a mediation model of self-esteem, spirituality, and psychological distress
Source: Front Sports Act Living. 2026 Apr 17;8:1782014. doi: 10.3389/fspor.2026.1782014 (PMC13133043; doi:10.3389/fspor.2026.1782014)
Supplement: Supplementary Appendix A — Uniqueness and item representation analysis of the 25 items of the Arabic version of the Ironson-Woods Spirituality/Religiousness Index (IWSRI). [file Table1.docx]

**Appendix A.** Component Matrix after Orthogonal Varimax Rotation of the Ironson-Woods Spirituality/Religiousness Index Questionnaire.

| Items | | Factor charges. | | | |  |
| --- | --- | --- | --- | --- | --- | --- |
|  |  | 1 | 2 | 3 | 4 | AVE |
| 1 | My beliefs give me a sense of peace. | 0.806 |  |  |  | 0.312 |
|  | تمنحني معتقداتي شعورًا بالسكينة والطمأنينة |  |  |  |  |  |
| 2 | My beliefs help me to know everything will be fine | 0.811 |  |  |  | 0.349 |
|  | تساعدني معتقداتي على الإحساس بأن الأمور ستسير على نحوٍ حسن |  |  |  |  |  |
| 3 | My beliefs give meaning to my life | 0.815 |  |  |  | 0.369 |
|  | تمنحني معتقداتي معنًى وغاية لحياتي |  |  |  |  |  |
| 4 | My beliefs help me to be relaxed. | 0.691 |  |  |  | 0.382 |
|  | تساعدني معتقداتي على الشعور بالهدوء والاسترخاء |  |  |  |  |  |
| 5 | My beliefs help me feel protected. | 0.612 |  |  |  | 0.383 |
|  | تجعلني معتقداتي أشعر بالحماية والأمان |  |  |  |  |  |
| 6 | My beliefs help me to feel I am not alone | 0.490 |  |  |  | 0.638 |
|  | تجعلني معتقداتي أشعر بأنني لست وحدي |  |  |  |  |  |
| 7 | My beliefs help me feel I have a relationship or a connection with a higher form of being | 0.728 |  |  |  | 0.355 |
|  | تجعلني معتقداتي أشعر بوجود صلة وروابط مع قوّة عليا |  |  |  |  |  |
| 8 | My beliefs help me be less afraid of death | 0.788 |  |  |  | 0.303 |
|  | تساعدني معتقداتي على تقليل خوفي من الموت |  |  |  |  |  |
| 9 | I believe my soul will live on in some form after my body dies | 0.836 |  |  |  | 0.270 |
|  | أؤمن بأن روحي ستستمر في الوجود بشكلٍ ما بعد وفاة الجسد |  |  |  |  |  |
| 10 | I believe God created all things in the universe |  | 0.844 |  |  | 0.275 |
|  | أؤمن بأن الله خلق كل الموجودات في الكون |  |  |  |  |  |
| 11 | God will not turn his back on me no matter what I do |  | 0.680 |  |  | 0.398 |
|  | أؤمن بأن الله لا يتخلى عنّي مهما حدث |  |  |  |  |  |
| 12 | When I am ill, God gives me courage to cope with my illness |  | 0.692 |  |  | 0.440 |
|  | عندما أمرض، تمنحني معتقداتي القوة والشجاعة لمواجهة المرض |  |  |  |  |  |
| 13 | When I am ill, God will answer my prayers for a recovery |  | 0.666 |  |  | 0.376 |
|  | عندما أمرض، أؤمن بأن الله يستجيب دعائي بالشفاء |  |  |  |  |  |
| 14 | My beliefs are very influential in my recovery when I am ill |  | 0.687 |  |  | 0.403 |
|  | تلعب معتقداتي دورًا مهمًّا في شفائي عندما أمرض |  |  |  |  |  |
| 15 | When I am ill, my faith gives me optimism that I will recover |  | 0.848 |  |  | 0.285 |
|  | حين أكون مريضًا، يمنحني إيماني تفاؤلًا وثقة بأنني سأتعافى |  |  |  |  |  |
| 16 | I attend religious services |  |  | 0.763 |  | 0.329 |
|  | أواظب على حضور الشعائر أو المناسبات الدينية |  |  |  |  |  |
| 17 | I participate in religious rituals (routine activities). |  |  | 0.807 |  | 0.345 |
|  | أشارك في الطقوس الدينية والأنشطة المرتبطة بها |  |  |  |  |  |
| 18 | I pray or meditate to get in touch with God. |  |  | 0.725 |  | 0.399 |
|  | أصلّي أو أتأمّل للتقرّب من الله |  |  |  |  |  |
| 19 | I discuss my beliefs with others who share my belief |  |  | 0.776 |  | 0.389 |
|  | أناقش معتقداتي مع الآخرين الذين يشاركونني نفس الإيمان |  |  |  |  |  |
| 20 | My beliefs give me a set of rules I must obey |  |  | 0.758 |  | 0.328 |
|  | تمنحني معتقداتي مجموعة من القيم والمبادئ التي ألتزم بها في حياتي |  |  |  |  |  |
| 21 | My beliefs teach me to help other people who are in need |  |  |  | 0.726 | 0.280 |
|  | تعلّمني معتقداتي مساعدة الآخرين المحتاجين |  |  |  |  |  |
| 22 | My beliefs help me feel compassion/love/respect for others |  |  |  | 0.805 | 0.288 |
|  | تساعدني معتقداتي على تنمية مشاعر الرحمة والمحبة والاحترام تجاه الآخرين |  |  |  |  |  |
| 23 | I have a responsibility to help others |  |  |  | 0.622 | 0.333 |
|  | أشعر بأن لدي مسؤولية تجاه مساعدة الآخرين |  |  |  |  |  |
| 24 | My beliefs increase my acceptance and tolerance of others |  |  |  | 0.592 | 0.326 |
|  | تزيد معتقداتي من تقبّلي وتسامحي مع الآخرين |  |  |  |  |  |
| 25 | I feel I am connected to all humanity |  |  |  | 0.749 | 0.295 |
|  | أشعر بأنني مرتبطٌ بالإنسانية جمعاء |  |  |  |  |  |
| Extraction method: Principal component analysis.  Rotation method: Varimax with Kaiser normalization. AVE = Average Variance Extracted | | | | | | |
| a. Convergence of the rotation in 5 iterations. | | | | | | |
